# Process-Driven Acetate-Based Lipid Production by the Oleaginous Yeast *Lipomyces starkeyi*

**DOI:** 10.3390/microorganisms14030608

**Published:** 2026-03-09

**Authors:** Akihiro Ishioka, Prihardi Kahar, Tasuku Nagano, Noor-Afiqah Ahmad Zain, Yutaro Mori, Chiaki Ogino

**Affiliations:** 1 Department of Chemical Science and Engineering, Graduate School of Engineering, Kobe University, 1-1 Rokkodai-cho, Nada-Ku, Kobe 657-8501, Hyogo, Japan; 2084726t@gmail.com (A.I.); tasu.homa728914@au.com (T.N.); noorafiqah51@gmail.com (N.-A.A.Z.); yutaro.mori@hawk.kobe-u.ac.jp (Y.M.); 2 Graduate School of Science, Technology, and Innovation (STIN), Kobe University, 1-1 Rokkodai-cho, Nada-Ku, Kobe 657-8501, Hyogo, Japan; 3 Engineering Biology Research Center, Kobe University, 1-1 Rokkodai-cho, Nada-Ku, Kobe 657-8501, Hyogo, Japan; 4 Research Center for Membrane and Film Technology, Kobe University, Kobe 657-8501, Hyogo, Japan

**Keywords:** *Lipomyces starkeyi*, oleaginous yeast, acetate utilization, pH control, fed-batch cultivation, microbial lipid production, single-cell oil, weak acid metabolism

## Abstract

Oleaginous yeasts are promising microbial platforms for lipid production from non-conventional carbon sources; however, acetate utilization is frequently constrained by physiological limitations associated with culture pH. In this study, acetate utilization, biomass formation, and lipid production by *Lipomyces starkeyi* were investigated under flask and fed-batch cultivation to evaluate the influence of culture pH and pH control strategy. Statistically supported flask-scale experiments demonstrated that acetate concentration and cultivation time significantly affected acetate consumption, biomass formation, volumetric lipid concentration, and culture pH, with excessive acetate loading resulting in culture alkalization, incomplete substrate utilization, and reduced process performance. Although volumetric lipid concentration increased with increasing acetate concentration, lipid content and fatty acid composition remained unchanged, indicating that enhanced lipid production was primarily attributable to increased biomass formation rather than to changes in lipid biosynthesis. Fed-batch cultivation under different pH-control strategies provided qualitative insights into the relationships among pH regulation, acetate availability, and lipid accumulation under controlled fermentation conditions. While lipid accumulation was observed under both HCl-based and acetic acid-based pH control, differences in pH stability and cumulative acetate availability were associated with distinct patterns of lipid production. Collectively, these results identify culture pH as a critical physiological parameter influencing acetate utilization and lipid accumulation in *L. starkeyi* and suggest that coordinated pH control and carbon feeding strategies may improve the robustness of acetate-based lipid production processes. Further replicated fed-batch studies will be required to quantitatively validate these trends and support industrial applications.

## 1. Introduction

The aviation sector currently accounts for approximately 2% of global anthropogenic CO_2_ emissions and is projected to increase through 2040 due to rising air travel demand [1]. Owing to stringent requirements for energy density, safety, and long-range operation, large-scale electrification of aviation remains technically challenging. Consequently, liquid hydrocarbon fuels are expected to remain indispensable for aviation in the near future. In response to climate change mitigation efforts, the International Civil Aviation Organization (ICAO) has established ambitious targets to reduce net CO_2_ emissions from international aviation by approximately 50% relative to 2005 levels by mid-century [2].

Among the proposed mitigation strategies, sustainable aviation fuel (SAF) has been recognized as a feasible near- to mid-term option for reducing life-cycle greenhouse gas emissions without requiring substantial modifications to existing aircraft or fuel infrastructure [3,4]. Current SAF production pathways include hydroprocessed esters and fatty acids, Fischer–Tropsch synthesis from syngas, and alcohol-to-jet processes [5,6]. However, many of these routes rely on food-competitive feedstocks, limited waste resources, or carbon-intensive upstream processes, raising concerns regarding long-term scalability and sustainability. Accordingly, the development of alternative, carbon-neutral feedstocks compatible with established fuel upgrading technologies remains a critical challenge [7,8,9].

In this context, microbial lipids produced by oleaginous microorganisms have attracted increasing attention as alternative feedstocks for SAF production [10,11,12,13,14]. These lipids are compositionally similar to plant-derived oils and predominantly consist of C16–C18 fatty acids, which are well suited for hydroprocessing-based SAF production [15,16]. Compared with conventional oil crops, microbial lipid production offers several advantages, including independence from arable land, shorter production cycles, and the ability to utilize a wide range of non-food carbon sources [17,18,19]. Among oleaginous microorganisms, yeasts are particularly attractive owing to their robustness, tolerance to harsh cultivation conditions, and compatibility with industrial fermentation systems [20,21]. Under appropriate nutrient limitation, oleaginous yeasts can accumulate lipids exceeding 50–70% of dry cell weight, enabling high volumetric lipid productivity [22,23].

Acetate has recently emerged as a promising unconventional carbon source for sustainable bioprocesses because it can be produced directly from CO_2_ via electrochemical or electrobiochemical conversion using renewable electricity [24,25]. In such systems, captured CO_2_ is reduced to acetic acid or acetate salts, converting gaseous carbon into a liquid, storable, and transportable carbon carrier. Unlike sugars derived from biomass, acetate does not compete with food resources and can be supplied directly to microbial cultivation systems without extensive pretreatment. From a metabolic perspective, acetate is assimilated via acetyl-CoA, a central metabolic intermediate and a direct precursor of fatty acid biosynthesis, making acetate an attractive substrate for microbial lipid production.

Despite these advantages, acetate-based microbial cultivation presents distinct process-level challenges. In practical fermentation systems, acetate is often supplied as sodium acetate or other acetate salts. During acetate uptake, counterions accumulate in the culture medium, leading to progressive alkalization. Elevated extracellular pH disrupts intracellular pH homeostasis and the proton motive force, impairing ATP generation and reducing metabolic activity. Such energy stress is particularly detrimental to lipid biosynthesis, which requires high fluxes of ATP and NADPH. In addition, alkaline conditions promote the conversion of ammonium ions to ammonia, thereby inhibiting key enzymes involved in central metabolism and lipid accumulation.

Previous studies have shown that uncontrolled pH drift during acetate utilization leads to growth inhibition, incomplete substrate utilization, and reduced volumetric lipid concentration in oleaginous microorganisms [26,27]. Although buffering strategies can partially mitigate pH fluctuations, their effectiveness is limited during continuous acetate uptake, especially at elevated substrate concentrations [28]. These findings indicate that pH imbalance represents a fundamental process bottleneck rather than a secondary operational issue in acetate-based microbial lipid production.

*Lipomyces starkeyi* is a non-conventional oleaginous yeast that has been extensively studied for its exceptional lipid accumulation capacity, often exceeding 60–70% of dry cell weight under nitrogen-limited conditions [29,30]. This yeast predominantly produces C16–C18 fatty acids suitable for SAF upgrading and exhibits tolerance to inhibitory compounds while also utilizing volatile fatty acids, including acetate [20,31]. Although several studies have reported lipid production by *L. starkeyi* from acetate or acetate-containing substrates [29,32,33], most were conducted at flask scale or focused primarily on metabolic outcomes rather than on process limitations.

Consequently, the interrelationship among acetate concentration, pH fluctuations, and cultivation mode remains insufficiently understood, particularly from a scalable bioprocess perspective. Systematic evaluation of pH control strategies under controlled bioreactor operation is therefore essential to translate the metabolic potential of *L. starkeyi* into an efficient acetate-based lipid production process.

In this study, we aimed to develop an effective acetate-based lipid production strategy using *L. starkeyi*, with particular emphasis on identifying and overcoming pH-related bottlenecks. Flask cultivation experiments were first conducted to assess acetate utilization and to elucidate limitations arising from acetate-induced pH drift. Subsequently, a pH-controlled bioreactor strategy was implemented to validate acetate assimilation and lipid production under controlled bioreactor conditions. By treating culture pH as a central process design variable rather than merely an operational parameter, this work provides process-oriented insights into acetate utilization by *L. starkeyi* and demonstrates the potential to convert CO_2_-derived acetate into microbial lipids for sustainable aviation fuel production.

## 2. Materials and Methods

### 2.1. Microorganism and Physiological Rationale for Strain Selection

The yeast strain *L. starkeyi* NBRC10381 used in this study was selected from the yeast library provided by NITE Biological Resource Center (NBRC) of the National Institute of Technology and Evaluation, Japan, based on its high lipid accumulation capacity, robust growth under nitrogen-limited conditions, and ability to utilize a wide range of carbon sources, including volatile fatty acids. Hereafter, this strain is referred to as *L. starkeyi* unless otherwise specified. Previous studies [29,30] have reported lipid contents exceeding 60–70% of dry cell weight for *L. starkeyi* under appropriate cultivation conditions, making it a suitable microbial chassis for lipid-based bioprocess development.

From a physiological perspective, *L. starkeyi* exhibits highly active acetyl-CoA metabolism and efficient fatty acid biosynthetic pathways, both of which are essential for acetate-based lipid production. In addition, this yeast shows tolerance to inhibitory compounds and osmotic stress, characteristics that are advantageous for cultivation on non-conventional substrates such as acetate. These features motivated the use of *L. starkeyi* as a model organism for investigating acetate-based lipid production under controlled bioprocess conditions.

### 2.2. Media Composition, Inoculum Preparation, and Cultivation Start-Up

For strain maintenance, *L. starkeyi* was cultivated on yeast extract–peptone–dextrose (YPD) agar plates containing 10 g L^−1^ yeast extract, 20 g L^−1^ peptone, 20 g L^−1^ glucose, and 15 g L^−1^ agar. Plates were incubated at 30 °C for 48–72 h and subsequently stored at 4 °C until use. The first preculture was initiated directly from glycerol stocks prepared from the maintenance plates. Cells were cultivated in a medium containing 10 g L^−1^ yeast extract, 20 g L^−1^ peptone, and 100 g L^−1^ glucose using 100 mL Erlenmeyer flasks with a working volume of 12 mL at 30 °C and 190 rpm (orbital shaking) for 3 days. Subsequently, a second preculture was conducted in a glucose-free medium consisting of 10 g L^−1^ yeast extract and 20 g L^−1^ peptone using 500 mL Sakaguchi flasks with a working volume of 120 mL at 30 °C and 120 rpm (reciprocal shaking) for 2–3 days. In both preculture steps, ampicillin and kanamycin were added as antibiotics at final concentrations of 200 mg L^−1^ and 40 mg L^−1^, respectively.

A nitrogen-limited minimal medium (−NMM) was used for all lipid production experiments. The −NMM consisted of a defined mineral medium containing sodium acetate at specific concentrations (for flask cultivation, 0 mM, 30 mM, 60 mM, 100 mM, and 200 mM, for bioreactor cultivation, 200 mM) for each experiment, a limited nitrogen supply, and essential macro- and micronutrients. Nitrogen sources included yeast extract (1.5 g L^−1^) and ammonium sulfate {(NH_4_)_2_SO_4_, 0.25 g L^−1^}, where yeast extract served as a source of organic nitrogen and B-complex vitamins to support initial cell growth, while ammonium sulfate was used at a growth-limiting concentration to promote lipid accumulation. The medium further contained MgSO_4_·7H_2_O (1.5 g L^−1^), phosphate buffer components KH_2_PO_4_ (7 g L^−1^) and Na_2_HPO_4_ (4 g L^−1^), and a trace element solution comprising FeSO_4_·7H_2_O (0.08 g L^−1^), ZnSO_4_·7H_2_O (0.01 g L^−1^), CaCl_2_·2H_2_O (0.1 g L^−1^), MnSO_4_·4H_2_O (0.1 g L^−1^), CuSO_4_·5H_2_O (0.002 g L^−1^), and CoCl_2_·6H_2_O (0.002 g L^−1^).

All medium components were sterilized by autoclaving at 121 °C for 20 min, except for (NH_4_)_2_SO_4_, phosphate buffer components, and other heat-labile constituents, including antibiotics and trace element solutions, which were sterilized by membrane filtration (0.22 μm) and aseptically added to the medium after cooling. To prevent bacterial contamination, ampicillin (200 mg L^−1^) and kanamycin (40 mg L^−1^) were added to the medium immediately before cultivation.

Cells from precultures were harvested by centrifugation at 6000× *g* for 5 min, washed with sterile distilled water to remove residual nutrients, and resuspended in −NMM. The main cultures were inoculated at an initial optical density (OD_600_) of 10, as specified in the experimental design. This relatively high initial cell density was selected to minimize lag-phase effects and to enable focused analysis of substrate utilization, pH dynamics, and lipid accumulation under nitrogen-limited conditions [34].

### 2.3. Flask Cultivation Experiments

Flask cultivation experiments were carried out in 500 mL Sakaguchi flasks with a working volume of 120 mL containing nitrogen-limited minimal medium (−NMM). Cultures were incubated at 30 °C and 120 rpm (reciprocal shaking) to maintain aerobic conditions, using Sakaguchi flasks to enhance oxygen transfer throughout cultivation. Flask cultures were primarily used in screening experiments to evaluate carbon-source utilization, substrate-concentration effects, and culture pH dynamics under uncontrolled conditions. Samples were withdrawn at regular intervals to determine carbon-source concentrations, dry cell weight (DCW), lipid content, and culture pH.

Carbon-source utilization was assessed by supplying sodium acetate as the sole carbon source. Control cultures without an added carbon source were included for comparison. These experiments were designed to distinguish acetate assimilation and to evaluate its suitability for supporting growth and lipid production by *L. starkeyi*.

The effect of acetate concentration on growth and lipid production was evaluated by conducting flask cultivations with initial sodium acetate concentrations ranging from 30 to 200 mM. These concentrations were selected to assess substrate tolerance, acetate consumption capacity, and the relationship between acetate availability and lipid accumulation, as well as to simulate conditions relevant to fed-batch or high-density cultivation. Culture performance was evaluated based on acetate consumption profiles, DCW, volumetric lipid concentration, and temporal changes in culture pH.

### 2.4. Bioreactor Cultivation and Process Control Strategy

Bioreactor cultivations were performed using laboratory-scale stirred-tank bioreactors with a working volume of 1.0 L. Cultures were operated at 30 °C with an initial agitation speed of 300 rpm and an aeration rate of 1.5 vvm. Dissolved oxygen (DO) concentration was continuously monitored using a polarographic DO probe and recorded in ppm via the bioreactor controller. Agitation speed was automatically adjusted between 300 and 600 rpm as required to maintain the DO concentration above 3.5 ppm.

Fed-batch cultivations were conducted in a 2 L bioreactor (ABLE Biott Co., Ltd., Tokyo, Japan) to evaluate acetate-based lipid production under two distinct pH-control strategies. A nitrogen-limited minimal medium (−NMM) was used as the basal medium and supplemented with 200 mM sodium acetate as the sole carbon source. Ampicillin and Kanamycin were added as antibiotics. Precultured cells were harvested as described above, concentrated, and inoculated into the bioreactor to achieve an initial optical density (OD) of 6.5–7.0.

To mitigate pH drift associated with acetate assimilation and sodium accumulation, culture pH was maintained between 5.5 and 6.5 using either 5 M hydrochloric acid (HCl) or 5 M acetic acid, depending on the experimental conditions. pH control was initiated after 12 h of cultivation and maintained thereafter.

In the first condition, pH was regulated using 5 M HCl to suppress alkalization during acetate assimilation, with the objective of achieving complete acetate consumption and enhanced lipid production. Under these conditions, carbon source feeding for additional sodium acetate was provided via a DO-stat strategy. A 3 M sodium acetate solution was automatically supplied when the dissolved oxygen (DO) concentration exceeded 5.0 ppm. The increase in DO was interpreted as an indicator of acetate depletion and therefore served as the trigger for carbon source feeding, restoring the acetate concentration to approximately 200 mM.

In the second condition, pH was controlled with 5 M acetic acid, which served as both a pH-adjusting agent and a carbon source. This strategy enabled continuous acetate supply by regulating the culture pH to the target range of 5.5–6.5. Aeration and agitation control parameters were identical to those used in the HCl-controlled condition.

For sampling, approximately 15 mL of culture broth was withdrawn at designated time points, and 10 mL was transferred to a pre-weighed 15 mL centrifuge tube. Samples were centrifuged at 3500 rpm for 5 min using a swing-rotor centrifuge. The supernatant was collected for analysis of residual carbon-source concentration by high-performance liquid chromatography (HPLC). The cell pellet was resuspended in deionized water and centrifuged again under the same conditions. After removal of the supernatant, the pellet was freeze-dried, and the dry cell weight (DCW) was determined gravimetrically as the difference between the pre-determined empty tube weight and the weight of the tube containing the freeze-dried biomass.

### 2.5. Analytical Methods

Concentrations of acetate in culture supernatants were determined by high-performance liquid chromatography (HPLC) equipped with a refractive index detector (RID-10A, Shimadzu, Kyoto, Japan). A Coregel-87H column (7.8 mm ID × 300 mm, Transgenomic Inc., New Haven, CT, USA) was used at 80 °C with 5 mM sulfuric acid as the eluent at a flow rate of 0.6 mL/min for 40 min. Before analysis, samples were filtered through 0.22 μm membrane filters.

Dry cell weight (DCW) was determined by collecting known volumes of culture broth, washing the harvested cells with distilled water to remove residual medium components, and freeze-drying the biomass. DCW values were used to calculate biomass concentration and lipid content on a mass basis.

Lipid production was quantified gravimetrically using a modified Folch extraction method following previously reported protocols [30,34] with minor modifications. For flask cultures, lipid extraction was performed using biomass harvested from approximately 10 mL of culture broth, concentrated by swing-out rotor centrifugation at 3500× *g* for 5 min, transferred into Corning tubes, and freeze-dried prior to lipid extraction. For bioreactor cultivations, freeze-dried biomass (15 mg) obtained after sampling was used directly. Zirconia beads (0.6 mm in diameter) and 1.0 mL of Folch solvent (chloroform: methanol = 2:1, *v*/*v*) were added to each tube. Cell disruption was performed using a Shakemaster Neo (Biomedical Science Co., Tokyo, Japan) for six 5-min cycles. The disrupted samples were subsequently incubated overnight at 25 °C and 1000 rpm in a Maximizer shaker to facilitate lipid extraction. After incubation, samples were centrifuged at 14,000 rpm for 2 min, and the supernatant was transferred to clean microcentrifuge tubes. To induce phase separation, 200 µL of 9 g L^−1^ sodium chloride solution was added to each tube, followed by vortex mixing and centrifugation at 14,000 rpm for 2 min. After separation into two phases, the lower organic phase was carefully collected in its entirety and transferred to pre-weighed microcentrifuge tubes. The solvent was evaporated under a chemical fume hood, followed by complete solvent removal in an oven at 80 °C. After cooling to room temperature, the tubes were weighed, and the lipid mass was calculated as the difference between the final weight and the weight of the empty tube. The extraction procedure, from the cell-disruption step onward, was repeated twice for each sample, and the total lipid extract was used to calculate lipid yield. Lipid analysis was performed in biological triplicates to ensure analytical robustness.

Lipid yield, lipid content, and volumetric lipid concentration were calculated using Formulas (1), (2), and (3), respectively.

(1) $$Lipid\;yield\;\left(w/w\;\mathrm{DCW}\right)=Weight\;of\;extracted\;lipid\;(g)/Weight\;of\;dry\;cells\;(g)$$

(2) $$Lipid\;content\left(\%,\;w/w\right)=Lipid\;yield\times 100\%$$

(3) $$Volumetric\;lipid\;concentration{\;(g\;L}^{-1})={Dry\;cell\;weight\;(g\;L}^{-1})\times Lipid\;content\;(\%,\;w/w)/100$$

Fatty acid composition was determined by fatty acid methyl ester (FAME) analysis. FAMEs were prepared from lipids extracted from freeze-dried biomass by transesterification using a commercial FAME kit (Nacalai Tesque, Kyoto, Japan) according to the manufacturer’s instructions. The resulting FAMEs were dissolved in hexane and analyzed by gas chromatography–mass spectrometry (GC–MS) using a QP-2010 system (Shimadzu, Kyoto, Japan). Individual fatty acids were identified by their mass spectra and retention times and quantified using absolute calibration curves constructed from a standard mixture (SUPELCO 37 FAME Mix; Sigma–Aldrich, St. Louis, MO, USA). Chromatographic separation was performed on a DB-23 capillary column (60 m × 0.25 mm i.d., 0.25 μm film thickness; Agilent Technologies, Santa Clara, CA, USA) using helium as the carrier gas. Samples (1 μL) were injected in split mode with the injector temperature set at 250 °C. The oven temperature program was as follows: 50 °C for 1 min, ramped to 175 °C at 25 °C min^−1^, then to 230 °C at 4 °C min^−1^, and held for 6 min. The MS detector temperature was set to 250 °C, and mass spectra were acquired in scan mode over an m/z range of 40–500.

Intracellular lipid accumulation was visualized by fluorescence microscopy after staining cells with BODIPY 493/503. Stained cells were imaged under appropriate excitation and emission settings to qualitatively confirm lipid droplet formation.

### 2.6. Statistical Analysis and Data Reproducibility

Flask cultivation experiments and bioreactor experiments were performed in biological triplicate. For flask cultivations, data are presented as mean values with corresponding standard deviations. Statistical analyses were performed using XLSTAT (version 2025.2.0) and included one-way or two-way analysis of variance (ANOVA), as appropriate, to evaluate the effects of acetate concentration, cultivation time, and pH control strategy. When ANOVA indicated significant differences, post hoc multiple comparisons were conducted using Tukey’s honestly significant difference (HSD) test. Differences were considered statistically significant at *p* < 0.05. The reproducibility of key observations—including acetate consumption profiles, pH control effects, biomass formation, and lipid accumulation—was confirmed through independent cultivation experiments.

## 3. Results

### 3.1. Carbon Source Utilization by Lipomyces starkeyi Under Flask Cultivation

**Figure 1:**
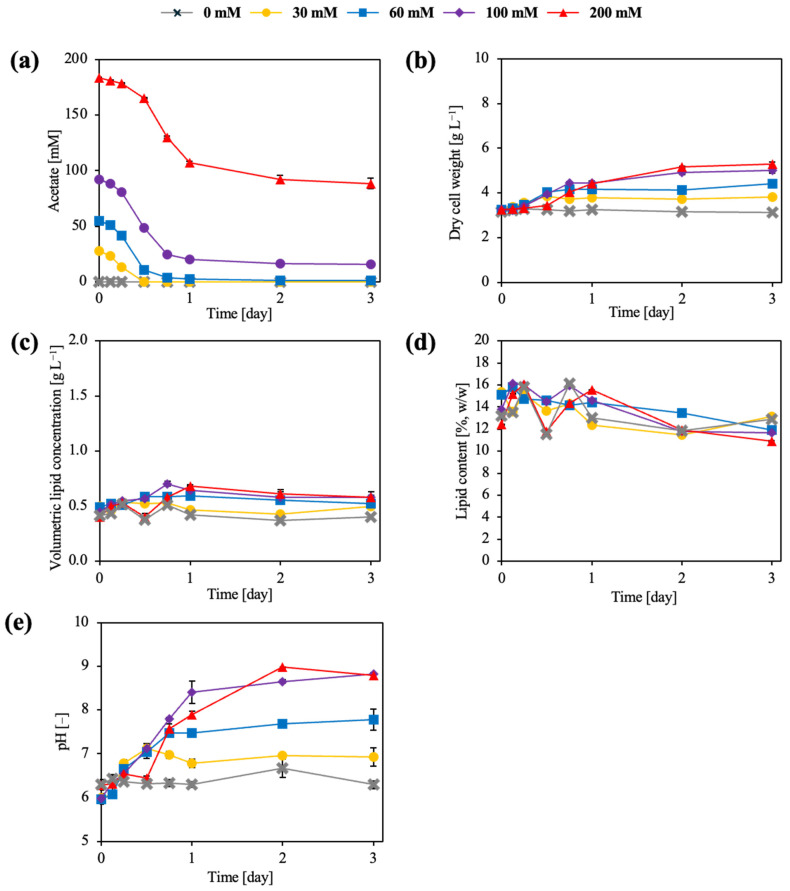
Effects of cultivation conditions on acetate utilization, biomass formation, lipid accumulation, and culture pH in *L. starkeyi*. (**a**) Time-course profiles of acetate consumption. (**b**) Biomass formation is expressed as dry cell weight (DCW). (**c**) Time-course profiles of lipid concentration (volumetric). (**d**) Lipid accumulation profiles. (**e**) Culture pH profiles during cultivation. Data are shown as mean values of biological replicates, with error bars indicating standard deviations. Symbols represent different initial acetate concentrations, as indicated in the legend.

Acetate utilization during flask cultivation was significantly affected by sodium acetate concentration and cultivation time (Figure 1), with a significant interaction between these factors (two-way ANOVA, *p* < 0.001). At 30 and 60 mM, acetate was almost completely consumed within 24 h, and residual concentrations did not differ significantly between these conditions at later time points (Tukey’s test, *p* > 0.05). Efficient acetate assimilation at moderate concentrations is consistent with the established capacity of oleaginous yeasts to channel acetate into acetyl-CoA-centered lipid metabolism under non-inhibitory conditions [32,33]. In contrast, cultures supplemented with 100 mM acetate exhibited incomplete consumption, with approximately 15–20 mM remaining at 72 h, which was significantly higher than in the 30 and 60 mM conditions (Tukey’s test, *p* < 0.01). Acetate utilization was most strongly inhibited at 200 mM, where more than 40% of the initial acetate remained unconsumed at 72 h (Tukey’s test, *p* < 0.001), in agreement with previous reports describing physiological constraints under excessive acetate loading [33,34]. Although nitrogen depletion can reduce carbon assimilation in oleaginous yeasts, the present observations are more consistent with pH-induced limitation of acetate utilization. During acetate assimilation, progressive alkalization of the culture medium was observed, which likely reduced the fraction of undissociated acetic acid available for transport across the plasma membrane. Elevated pH conditions are known to impair acetate uptake efficiency and impose intracellular pH stress, thereby limiting both growth and lipid biosynthesis despite the continued presence of carbon substrate. Therefore, residual acetate accumulation in the late cultivation stage is primarily due to process-driven pH effects rather than to nitrogen exhaustion alone.

As shown in Figure 1, biomass formation was significantly influenced by acetate concentration and cultivation time (two-way ANOVA, *p* < 0.001). In the absence of acetate, dry cell weight (DCW) remained low throughout cultivation, reaching approximately 3.1 g L^−1^ at 72 h. Supplementation with 60 and 100 mM acetate resulted in significantly higher DCW values (approximately 4.5–5.0 g L^−1^) compared with the acetate-free condition (Tukey’s test, *p* < 0.01), while the highest DCW was observed at 200 mM acetate (approximately 5.3 g L^−1^; Tukey’s test, *p* < 0.05). These results are consistent with previous studies demonstrating that acetate supports biomass formation in oleaginous yeasts by efficiently entering central carbon metabolism [35,36].

Volumetric lipid concentration (g L^−1^) was significantly affected by acetate concentration and time (two-way ANOVA, *p* < 0.001). Cultures without acetate produced the lowest volumetric lipid concentration (approximately 0.40 g L^−1^), whereas supplementation with 60–100 mM acetate significantly increased lipid production to approximately 0.55–0.65 g L^−1^ (Tukey’s test, *p* < 0.01). The highest volumetric lipid concentration was obtained at 200 mM acetate (approximately 0.65–0.70 g L^−1^), which was significantly higher than that observed in the acetate-free condition (Tukey’s test, *p* < 0.001). In contrast, lipid content (% DCW) did not differ significantly among acetate concentrations or time points (two-way ANOVA, *p* > 0.05), remaining within a narrow range of approximately 12–15%. This decoupling between volumetric lipid concentration and lipid content reflects biomass-driven increases in total lipid production rather than enhanced intracellular lipid accumulation [37,38]. Although reduced biomass formation was associated with lower volumetric lipid concentration, this relationship alone does not explain the observed process behavior. Instead, the results indicate that cultivation conditions influenced metabolic carbon partitioning, thereby simultaneously constraining growth and lipid synthesis. These findings highlight that acetate-based lipid production is primarily governed by process-driven physiological regulation rather than biomass formation alone.

Culture pH was significantly affected by acetate concentration and cultivation time (two-way ANOVA, *p* < 0.001). In low-acetate cultures (0–30 mM), pH remained relatively stable, whereas cultures supplemented with ≥60 mM acetate exhibited progressive alkalization, reaching pH values of approximately 7.5–8.5 at 72 h, with the 200 mM condition approaching pH 9.0 (Tukey’s post hoc test, *p* < 0.001). This alkalization during acetate assimilation has been attributed to proton consumption associated with acetate uptake and its intracellular activation to acetyl-CoA, a phenomenon that is further exacerbated when acetate is supplied as sodium acetate due to the accumulation of sodium ions in the medium [39]. Such alkalization represents a major physiological limitation for acetate utilization under non-pH-controlled cultivation conditions.

**Figure 2:**
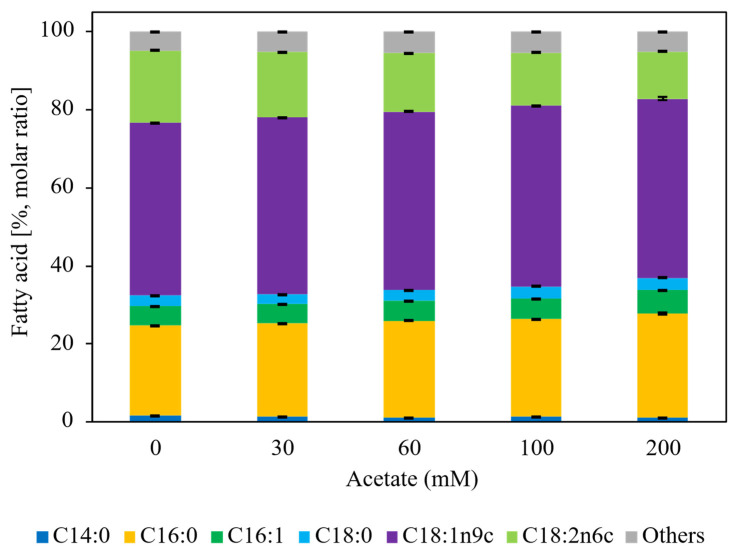
Fatty acid composition of intracellular lipids produced by *L. starkeyi* during flask cultivation with different sodium acetate concentrations at Day 3. Fatty acid distributions are expressed as molar percentages calculated from the molar ratios of individual fatty acids relative to total fatty acids and include oleic acid (C18:1 n-9), palmitic acid (C16:0), linoleic acid (C18:2), stearic acid (C18:0), and minor components. Data represent mean values with standard deviations from biological replicates. No significant differences in fatty acid composition were observed among acetate concentrations (one-way ANOVA, *p* > 0.05).

Figure 2 showcases that fatty acid composition at 72 h was not significantly affected by acetate concentration (one-way ANOVA, *p* > 0.05). Across all conditions, oleic acid (C18:1 n-9) was the dominant fatty acid, followed by palmitic, linoleic, and stearic acids, a profile characteristic of oleaginous yeasts cultivated on diverse carbon sources. This distribution is consistent with previous metabolic characterizations of *L. starkeyi*, indicating that acetate concentration influences lipid quantity but not lipid quality [38].

Collectively, these results demonstrate that acetate concentration alone does not dictate lipid production performance during the acetate-based cultivation of *L. starkeyi.* While previous studies have highlighted the metabolic versatility of *L. starkeyi* and investigated lipid accumulation from diverse carbon sources, most reports have focused primarily on strain physiology or substrate optimization under relatively stable cultivation conditions [29,40,41]. Consequently, the process-level physiological mechanisms responsible for instability during acetate fermentation have remained insufficiently clarified. The present study addresses this gap by identifying culture pH elevation as a primary process bottleneck linking acetate assimilation to impaired substrate utilization and reduced lipid productivity.

Systematic analysis of acetate consumption, biomass formation, and extracellular pH dynamics revealed that progressive alkalization accompanying acetate uptake constitutes a dominant physiological constraint limiting effective carbon assimilation. Rather than functioning as a secondary operational parameter, culture pH directly governs substrate availability by altering acetate dissociation equilibria, transport efficiency, and intracellular metabolic activity. The observation that lipid content and fatty acid composition remained largely unchanged across acetate concentrations further indicates that the decline in volumetric lipid productivity at high acetate loading originates from process-level physiological limitations rather than intrinsic alterations in lipid biosynthesis capacity.

### 3.2. Effect of pH Control Strategy on Acetate Utilization and Lipid Production

Acetate consumption during fed-batch cultivation was strongly influenced by the pH control strategy, consistent with the pH-dependent acetate utilization observed in flask cultivation. In flask experiments, acetate consumption was significantly affected by acetate concentration and cultivation time, with a significant interaction between these factors (two-way ANOVA, *p* < 0.001). Incomplete utilization at high acetate concentrations coincided with culture alkalization (Figure 1).

**Figure 3:**
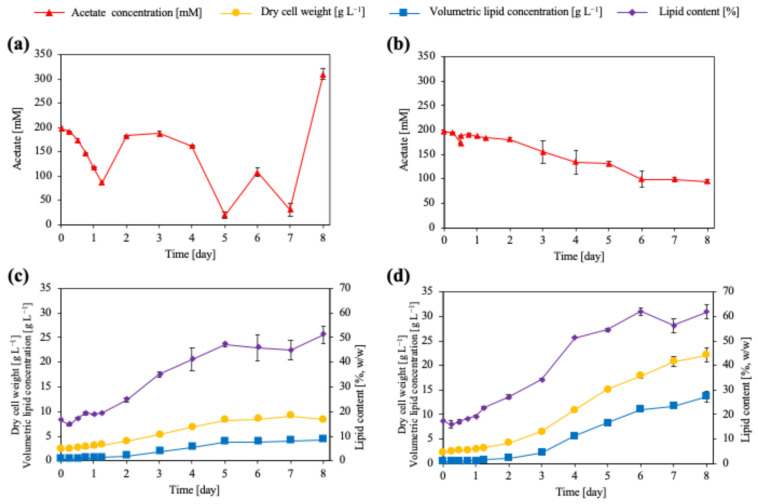
Time courses of fermentation parameters during fed-batch cultivation of *L. starkeyi* under different pH control strategies. (**a**) Residual acetate concentration which pH was controlled using 5 M hydrogen chrolide (HCl), (**b**) residual acetate concentration which pH was controlled using 5 M acetic acid, (**c**) dry cell weight, lipid concentration (volumetric), and lipid content during fed-batch cultivation (HCl), and (**d**) dry cell weight, lipid concentration (volumetric), and lipid content during fed-batch cultivation (acetic acid). Data are shown as mean values of biological replicates, with error bars indicating standard deviations. Symbols represent different fermentation parameters, as indicated in the legend.

In fed-batch cultivation, where acetate concentration was regulated by feeding and culture pH was continuously controlled, distinct acetate concentration profiles were observed depending on the titrant used (Figure 3). Under pH control with 5 M HCl, the acetate concentration exhibited pronounced temporal fluctuations, characterized by rapid depletion followed by transient re-accumulation during the early cultivation phase. In contrast, pH control with 5 M acetic acid yielded a comparatively stable acetate profile, with acetate concentrations decreasing gradually. This decrease is due to the gradual depletion of sodium ions caused by sampling. This stabilized acetate availability closely parallels the flask-cultivation results observed at moderate acetate concentrations (30–60 mM), where efficient acetate utilization occurred without excessive pH elevation. Collectively, these results support the conclusion that acetate availability and culture pH function as tightly coupled determinants governing acetate assimilation during fed-batch cultivation. Furthermore, acetic acid used for pH control functioned not only as a titrant but also as an additional assimilable carbon source. Periodic addition of acetic acid inevitably increased the total acetate supply during fed-batch cultivation. While this supplementary carbon input partially contributed to enhanced biomass formation and lipid accumulation, the observed improvement cannot be explained solely by increased carbon availability. Instead, stabilization of culture pH prevented excessive alkalization, thereby maintaining favorable acetate transport equilibria and metabolic activity. These results indicate that acetic acid simultaneously serves as a pH regulator and a carbon substrate, with pH stabilization as the dominant process factor, enabling efficient acetate assimilation and lipid production.

Dry cell weight (DCW) responses in fed-batch cultivation were also consistent with trends observed in flask cultures. In flask cultivation, DCW was significantly enhanced by acetate supplementation (two-way ANOVA, *p* < 0.001), but biomass formation plateaued or declined under conditions associated with pH elevation. Similarly, in fed-batch cultivation, DCW increased under both pH control strategies, but the magnitude and temporal stability differed significantly. Under HCl-controlled conditions, DCW increased gradually to approximately 8–9 g L^−1^ by day 7 and subsequently declined during the late cultivation phase, consistent with the biomass decrease observed in flask cultures following acetate depletion or alkalization. In contrast, acetic-acid-controlled cultures exhibited sustained biomass accumulation, reaching approximately 22.1 g L^−1^ by Day 8. The substantially higher biomass obtained under acetic acid pH control is consistent with continuous acetate availability and mitigation of the pH-associated growth limitation identified in flask cultivation.

Lipid production in fed-batch cultivation reflected the same biomass-driven behavior observed in flask experiments. In flask cultures, volumetric lipid concentration (g L^−1^) increased significantly with acetate concentration and time (two-way ANOVA, *p* < 0.001), whereas lipid content (%, *w*/*w*) did not differ significantly among conditions (*p* > 0.05), indicating that increased lipid production primarily reflected increased biomass formation. In fed-batch cultivation, volumetric lipid concentration was again strongly dependent on the pH control strategy. Under HCl-based pH control, volumetric lipid concentration increased modestly to approximately 3–4 g L^−1^ and subsequently remained at the same level. Notably, the total amount of acetate supplied during cultivation was lower under HCl-based pH control than under acetic acid-based pH control. This reduced cumulative acetate input may have contributed to the lower overall lipid production observed under the HCl-controlled condition. In contrast, acetic-acid-controlled fed-batch cultivation yielded substantially higher lipid levels, reaching approximately 13.7 g L^−1^ by Day 8. Unlike in flask cultivation, lipid content under acetic acid pH control increased to values exceeding 60% (*w*/*w*), indicating that sustained acetate supply under pH-controlled, carbon-excess conditions enabled both biomass accumulation and efficient lipid storage, consistent with previous reports on fed-batch oleaginous yeast systems. Lipid production using acetate as the sole carbon source in *L. starkeyi* has rarely been reported, and among reported lipid productions using acetate, this represents the highest value.

**Figure 4:**
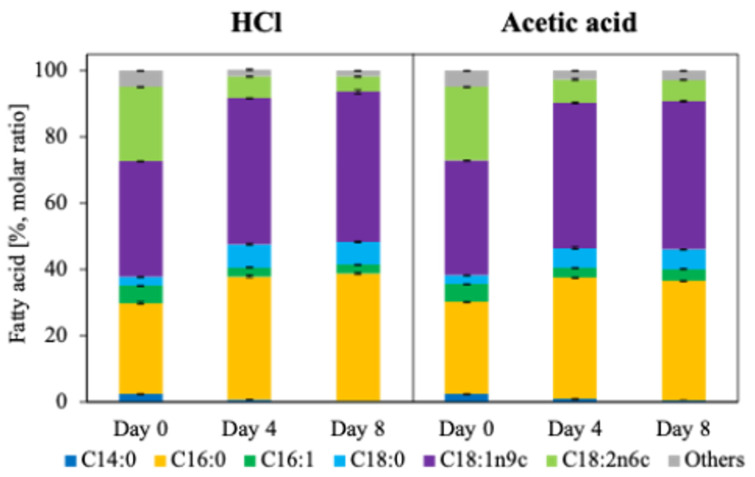
Time-course changes in fatty acid composition during fed-batch fermentation. Fatty acid profiles were determined at Day 0, Day 4, and Day 8 after the initiation of fed-batch cultivation and are expressed as relative percentages of total fatty acids. Culture pH was controlled using 5 M HCl (HCl condition) or 5 M acetic acid (acetic acid condition). Stacked bars represent the proportional distribution of individual fatty acids, including C14:0 (myristic acid), C16:0 (palmitic acid), C16:1 (palmitoleic acid), C18:0 (stearic acid), C18:1 (oleic acid), C18:2 (linoleic acid), and C18:3 (linolenic acid). The results illustrate condition- and time-dependent remodeling of fatty acid composition during fed-batch fermentation.

Fatty acid composition remained unchanged across cultivation modes and pH control strategies. In flask cultivation (Figure 2), fatty acid profiles were not significantly affected by acetate concentration (one-way ANOVA, *p* > 0.05), and the same compositional stability was observed in fed-batch cultivation (Figure 4). In all cases, oleic acid (C18:1 n-9) was the dominant fatty acid, followed by C16:0 (palmitic acid), C18:2 (linoleic acid), and C18:0 (stearic acid). However, temporal changes in fatty acid composition were observed during fed-batch cultivation. As fermentation progressed, the relative proportions of C16:0 and C18:1 increased. These fatty acids have chain lengths and saturation characteristics well-suited for sustainable aviation fuel (SAF) and biodiesel production. Therefore, the observed shift toward C16–C18 fatty acids represents a favorable compositional trend from an industrial fuel-production perspective.

Overall, alignment of flask and fed-batch results demonstrates that culture pH is a primary determinant of acetate utilization efficiency and lipid production by *L. starkeyi*. Flask cultivation revealed pH-associated limitations at high acetate concentrations, while fed-batch cultivation with acetic acid-based pH control effectively alleviated these constraints by stabilizing both pH and acetate availability. These statistically consistent observations across cultivation scales identify pH control strategy as a dominant process variable and provide a robust rationale for implementing acetic acid-based pH control in scalable acetate-driven lipid production processes.

### 3.3. Intracellular Lipid Accumulation Under Different pH Control Strategies

**Figure 5:**
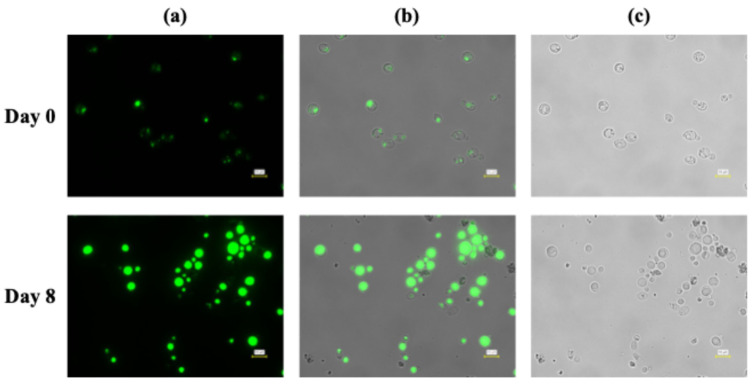
Fluorescence microscopy analysis of intracellular lipid accumulation in *L. starkeyi* during fed-batch cultivation with pH controlled using 5 M hydrogen chloride. Cells were sampled at Day 0 (upper panels) and Day 8 (lower panels). (**a**) Fluorescence images showing intracellular lipid droplets stained with BODIPY 493/503. (**b**) Overlay images of fluorescence and bright-field signals. (**c**) Corresponding bright-field images. Scale bars = 10 µm.

**Figure 6:**
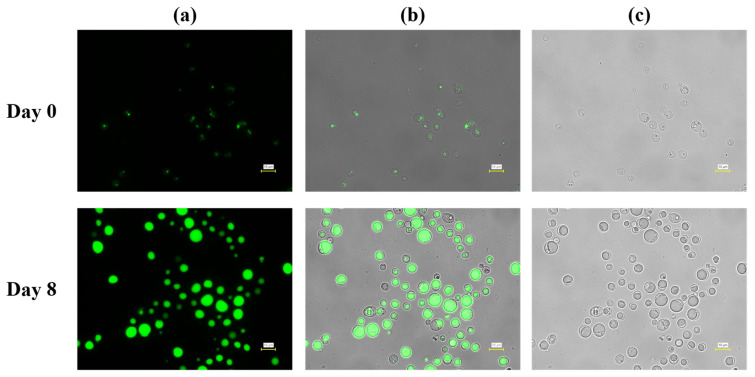
Fluorescence microscopy analysis of intracellular lipid accumulation in *L. starkeyi* during fed-batch cultivation with pH controlled using 5 M acetic acid. Cells were sampled at Day 0 (upper panels) and Day 8 (lower panels). (**a**) Fluorescence images showing intracellular lipid droplets stained with BODIPY 493/503. (**b**) Overlay images of fluorescence and bright-field signals. (**c**) Corresponding bright-field images. Scale bars = 10 µm.

Microscopic observations further corroborated the quantitative differences in lipid accumulation observed under the two pH control strategies during fed-batch cultivation. At Day 0, cells cultivated under both pH control conditions exhibited weak or minimal fluorescence signals, indicating low intracellular lipid levels at the onset of fed-batch operation (Figure 5 and Figure 6). Cells appeared relatively small and uniform, with only faint lipid-droplet signals detectable, consistent with the low lipid content measured at this stage and with previous reports on oleaginous yeasts before lipid induction.

After 8 days of cultivation under pH control using 5 M HCl, fluorescence microscopy revealed a clear increase in intracellular lipid droplets relative to Day 0 (Figure 5). Lipid droplets were visible in a substantial fraction of the cell population; however, their size and distribution were heterogeneous. Many cells contained multiple small to medium-sized droplets, while others exhibited weaker fluorescence signals. This heterogeneity is consistent with the moderate lipid content during the late phase of HCl-controlled fed-batch cultivation. Phase-contrast images confirmed intact cell morphology but also indicated variability in cell size, suggesting uneven lipid accumulation across the population, a phenomenon commonly reported in oleaginous yeasts under suboptimal or fluctuating carbon supply [42,43].

In contrast, cells cultivated under pH control with 5 M acetic acid exhibited markedly stronger and more uniform fluorescence signals at Day 8 (Figure 6). Large, well-defined lipid droplets occupied a substantial fraction of the intracellular volume in most cells, and fluorescence intensity was consistently higher than that observed under HCl pH control. Phase-contrast images showed enlarged cells with pronounced intracellular inclusions, consistent with extensive lipid storage. Notably, this enhanced intracellular lipid accumulation was observed during the mid-to-late fed-batch phase, indicating a shift in overall metabolic demand consistent with lipid storage-dominant physiology, as reported for oleaginous yeasts undergoing the transition from growth to storage lipid synthesis under nitrogen-limited, carbon-excess conditions [42].

Importantly, the qualitative differences observed by microscopy align with the statistically supported trends in volumetric lipid concentration and lipid content obtained from bulk measurements. Under HCl pH control, lipid accumulation was detectable but heterogeneous, whereas under acetic acid pH control, it was widespread and robust across the cell population. These observations support the conclusion that the superior lipid production performance under acetic acid pH control reflects enhanced intracellular lipid storage rather than analytical artifacts or sampling bias, consistent with prior fed-batch studies using acetate or other weak acids as carbon sources [37,43].

Together, the microscopy data support the conclusion that the pH control strategy influences lipid accumulation at the single-cell level during fed-batch cultivation. While both strategies enabled lipid droplet formation, acetic acid-based pH control promoted more homogeneous and extensive lipid accumulation, without apparent morphological abnormalities, consistent with its superior performance in biomass formation, volumetric lipid concentration, and lipid content.

## 4. Discussion

Fluctuations in culture pH exert a profound influence on microbial metabolism and have important implications for biotechnological applications, particularly lipid production from renewable carbon sources. *Lipomyces starkeyi* has attracted considerable attention as an oleaginous yeast capable of efficiently converting acetate into storage lipids, positioning it as a promising microbial platform for sustainable biodiesel and lipid-based bioprocesses [32,44,45]. As interest in acetate-based fermentation systems continues to grow, a detailed understanding of the metabolic pathways involved and the physiological constraints imposed by pH dynamics is essential for optimizing lipid synthesis at the industrial scale.

Acetic acid serves as a key carbon source for *L. starkeyi* and is assimilated primarily through metabolic routes involving the glyoxylate shunt and the tricarboxylic acid (TCA) cycle, ultimately generating acetyl-CoA as the central precursor for fatty acid biosynthesis. Efficient lipid accumulation, therefore, depends not only on acetate availability but also on environmental parameters that regulate intracellular metabolic fluxes, with culture pH being a particularly critical factor. Previous studies have demonstrated that maintaining an appropriate pH during fermentation significantly enhances acetate utilization and lipid production, especially under fed-batch conditions [44]. These findings support the view that pH control functions not merely as an auxiliary operational parameter but as an active determinant of carbon partitioning toward lipid synthesis.

Culture pH directly affects enzymatic activity, redox balance, and intracellular homeostasis in *L. starkeyi.* Variations in extracellular pH can alter the activity and regulation of key enzymes involved in lipid metabolism, such as acetyl-CoA synthetase and fatty acid synthase, thereby influencing both cell growth and lipid accumulation. Deviations from the optimal pH range have been shown to markedly reduce biomass formation and volumetric lipid concentration, underscoring the sensitivity of *L. starkeyi* to pH stress [45]. Both acidic and alkaline conditions can disrupt metabolic coordination, leading to inefficient acetate assimilation and reduced lipid productivity.

The importance of pH control becomes particularly evident when *L. starkeyi* is cultivated on lignocellulosic hydrolysates or other complex feedstocks, in which acetate is often accompanied by additional inhibitory compounds. In such systems, inadequate pH regulation can exacerbate physiological stress, further limiting lipid production [46]. Effective pH management mitigates the combined inhibitory effects of organic acids and hydrolysate-derived byproducts, enabling more robust metabolic performance and higher lipid accumulation rates [32,47]. Accordingly, controlled pH conditions have been widely reported to improve cell growth, acetate consumption, and volumetric lipid concentration while stabilizing fatty acid composition during cultivation [32,48].

In the present study, fed-batch cultivation under different pH-control strategies revealed distinct qualitative differences in acetate utilization and lipid accumulation behavior. Although lipid accumulation was observed under both HCl-based and acetic acid-based pH control, the overall volumetric lipid concentration was lower under HCl-based pH control. In this context, the reduced cumulative acetate availability under HCl-based pH control—where acetate was supplied solely as a carbon source rather than concurrently via pH regulation—likely contributed to the observed differences in lipid accumulation, in addition to pH-related physiological effects. These observations highlight the intrinsic coupling between pH control strategy and effective carbon supply in acetate-based lipid production processes, while also underscoring the need for cautious interpretation of fermenter-scale data.

At the molecular level, transcriptomic and systems-level analyses reported in the literature indicate that pH fluctuations can trigger extensive changes in gene expression during lipid accumulation, affecting pathways related to central carbon metabolism, stress response, and fatty acid synthesis [49]. While the present study does not directly address transcriptional regulation, the observed physiological trends are consistent with these reports and suggest that pH control may guide metabolic responses toward more efficient acetate utilization and lipid storage.

From an industrial perspective, effective pH management remains a critical determinant of the feasibility of *L. starkeyi*-based lipid production. Optimized pH control has been shown to improve substrate utilization efficiency, reduce process instability, and facilitate the use of acetate-rich and inhibitor-containing feedstocks derived from agricultural residues and organic waste streams [40,49,50]. Through replicated fed-batch experiments, the relationships at the process level were quantitatively verified, and their robustness was confirmed.

## 5. Conclusions

This study examined the role of culture pH in acetate utilization and lipid production by *L. starkeyi* under both flask and fed-batch cultivation conditions. Statistically supported flask-scale experiments demonstrated that acetate concentration and cultivation time significantly influenced acetate consumption, biomass formation, volumetric lipid concentration, and culture pH, with excessive acetate loading leading to culture alkalization, incomplete substrate utilization, and reduced process performance. Although volumetric lipid concentration increased with increasing acetate concentration, lipid content and fatty acid composition remained unchanged, indicating that enhanced lipid production was primarily associated with increased biomass formation rather than alterations in lipid biosynthetic patterns. Fed-batch cultivation under different pH control strategies provided qualitative insights into the relationships among pH regulation, acetate availability, and lipid accumulation under fully controlled fermentation conditions. While lipid accumulation was observed under both HCl-based and acetic acid-based pH control, differences in pH stability and cumulative acetate availability were associated with distinct lipid production behaviors, highlighting the intrinsic coupling between pH control strategy and effective carbon supply in acetate-based lipid production processes. Notably, the successful cultivation of *L. starkeyi* using acetic acid as the sole carbon source enabled lipid production reaching 13.7 g L^−1^ by Day 8. Collectively, these findings demonstrate that culture pH functions as a critical physiological determinant governing acetate assimilation and lipid accumulation in *L. starkeyi*, and they emphasize that coordinated pH regulation and carbon feeding strategies are essential for improving the stability and scalability of acetate-driven microbial lipid production processes.

## Data Availability

The data presented in this study are available in this article. Further inquiries can be directed to the corresponding authors.

## Abbreviations

The following abbreviations are used in this manuscript:

DO-stat	Dissolved oxygen–stat feeding strategy
FAMEs	Fatty acid methyl esters
GC–MS	Gas chromatography–mass spectrometry
HCl	Hydrochloric acid
HPLC	High-performance liquid chromatography
NBRC	NITE Biological Resource Center
OD_600_	Optical density at 600 nm
−NMM	Nitrogen-limited minimal medium
SAF	Sustainable aviation fuel
TCA cycle	Tricarboxylic acid cycle
